# A Novel High-Resolution Single Locus Sequence Typing Scheme for Mixed Populations of *Propionibacterium acnes In Vivo*

**DOI:** 10.1371/journal.pone.0104199

**Published:** 2014-08-11

**Authors:** Christian F. P. Scholz, Anders Jensen, Hans B. Lomholt, Holger Brüggemann, Mogens Kilian

**Affiliations:** Department of Biomedicine, Aarhus University, Aarhus, Denmark; St. Petersburg Pasteur Institute, Russian Federation

## Abstract

The Gram-positive anaerobic bacterium *Propionibacterium acnes* is a prevalent member of the normal skin microbiota of human adults. In addition to its suspected role in acne vulgaris it is involved in a variety of opportunistic infections. Multi-locus sequence-typing (MLST) schemes identified distinct phylotypes associated with health and disease. Being based on 8 to 9 house-keeping genes these MLST schemes have a high discriminatory power, but their application is time- and cost-intensive. Here we describe a single-locus sequence typing (SLST) scheme for *P. acnes*. The target locus was identified with a genome mining approach that took advantage of the availability of representative genome sequences of all known phylotypes of *P. acnes*. We applied this SLST on a collection of 188 *P. acnes* strains and demonstrated a resolution comparable to that of existing MLST schemes. Phylogenetic analysis applied to the SLST locus resulted in clustering patterns identical to a reference tree based on core genome sequences. We further demonstrate that SLST can be applied to detect multiple phylotypes in complex microbial communities by a metagenomic pyrosequencing approach. The described SLST strategy may be applied to any bacterial species with a basically clonal population structure to achieve easy typing and mapping of multiple phylotypes in complex microbiotas. The *P. acnes* SLST database can be found at http://medbac.dk/slst/pacnes.

## Introduction

*Propionibacterium acnes* is a Gram-positive anaerobic bacterium ubiquitously present in human sebaceous follicles of the skin [1], [2]. Although *P. acnes* is a commensal, it is most commonly known for its assumed role in the pathogenesis of acne vulgaris [3]–[5]. In addition, it has also been associated with infections in prosthetic joints [6], [7], the endodontium [8], eyes post surgically [9], lumbar discs [10], [11], the prostate [12], [13], and other tissues [14].

The dual role of the *P. acnes* as a health-associated bacterium and an opportunistic pathogen led to the assumption that certain strains may possess an elevated pathogenic potential. In agreement with this hypothesis, the population structure resolved by multi-locus sequence-typing (MLST) analyses revealed distinct health- and disease-associated lineages of *P. acnes*. Due to the relative sequence conservation of the genome, high-resolution typing required an MLST scheme based on nine loci (here designated MLST9) [15]. An alternative seven locus MLST scheme (MLST7) was introduced by [16]. A comparison of the two schemes favoured the MLST9 scheme based on higher resolution and congruency with a phylogenetic reference tree based on concatenated sequences of 76 housekeeping genes [17]. Subsequently, the MLST7 scheme was modified by substituting one of the loci with two new (MLST8) [18]. A recent publication by McDowell *et al*. described a 4-locus MLST (MLST4) based on the MLST8 scheme, with the aim to reduce time and cost of typing *P. acnes* strains [19]. An alternative approach was reported by Fitz-Gibbon *et al*. who used 16S rRNA gene ribotyping in a metagenomic approach to investigate the clonal diversity of *P. acnes* on the skin of acne patients and healthy individuals [20].

The mentioned MLST schemes are labour intensive and are not suitable for identification of multiple *P. acnes* phylotypes in sequence-based metagenomic studies. Sequencing of the 16S rRNA gene is cheaper and can in metagenomic studies identify bacterial taxa colonising the same sample site, but with limited resolution.

In this study we developed a single-locus sequence typing (SLST) scheme for *P. acnes* with a discriminatory power comparable to that of multi-locus approaches. The target locus was identified with a genome mining approach with reference to the genetic population structure of the species. This SLST approach provides a solution to the need for mapping of multiple phylotypes in complex microbial communities and may be applied to any bacterial species with a basically clonal population structure.

## Materials and methods

### Ethics statement

The Regional Danish Scientific Ethics Committee (N-20120050) approved the study, and written informed consent was obtained from the subject.

### Phylogenetic reference tree

**Figure 1:**
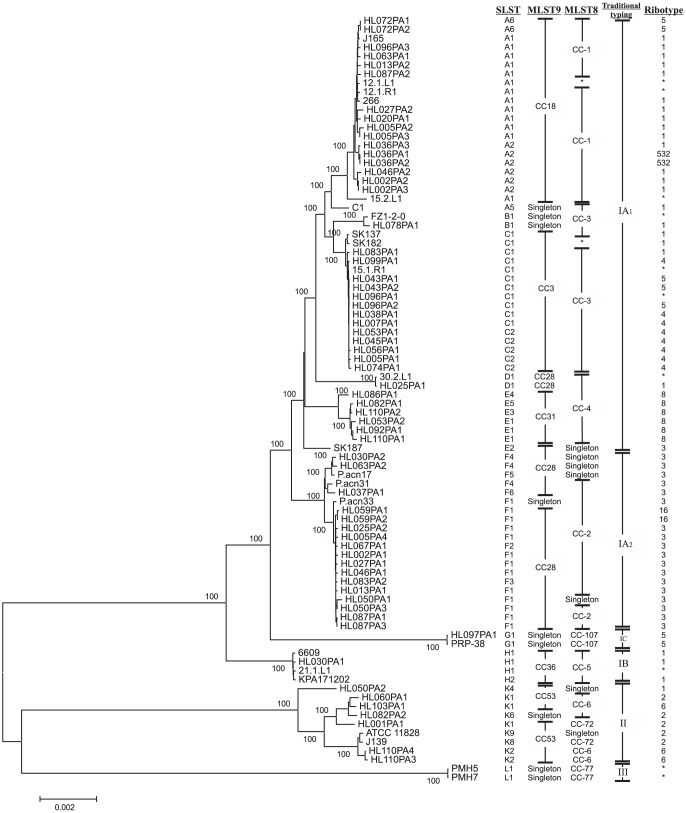
The SLST scheme discriminates the phylogenetic clusters of *P. acnes*. Minimum Evolution phylogenetic tree based on 86 complete and draft *P. acnes* genomes, generated in MEGA v. 5.22 and based on a 1,964,522-bp concatemer of core sequences. Only bootstrap values of 100% are shown. The leftmost column depicts the identified SLST type. Type designations according to MLST9 and MLST8 schemes and ribotyping are also given, in addition to the traditional type assignments based on *recA* and *tly* sequence analysis. * indicates unknown types.

A phylogenetic reference tree was constructed using shared core sequences of 86 *P. acnes* genomes of the 188 strains used in this study (Table S1). The core of the 86 genomes was obtained by splitting the genome sequence of the reference strain *P. acnes* KPA171202 into 500 bp fragments and aligning each fragment against all other genomes using blastn v. 2.2.28+ [21] with the following cut-off parameters: coverage > 90% and identity > 80%. Any fragment that did not yield a hit from all genomes was discarded, and the rest were aligned using Muscle v. 3.8.31[22] and concatenated into a 1,964,522 bp-sequence, hereafter referred to as the core genome. Using MEGA v. 5.2.2[23] we identified 107,397 single nucleotide polymorphisms (SNPs) and 8,100 gaps. A reference phylogenetic tree (Figure 1) was built in MEGA [23] using the Minimal evolution algorithm and 500 replication in the bootstrap test with the “complete deletion” option to exclude gaps from the analysis.

### Identification of SLST candidates

The strategy aimed at scanning the entire genome to identify an approximately 500 bp DNA fragment suitable for SLST. This sequence length was chosen to ensure that overlapping reads from forward and reverse Sanger sequencing would result in high quality sequences.

A sliding window of 500 bp running along the genome (step size +1 bp) ensured that all possible candidates were evaluated. For each base pair step an alignment of the fragment to the rest of the genomes was done. The corresponding sequences from the other genomes were identified by blastn in the BLAST+ suite [21] and aligned with Muscle [22]. These blastn searches were made in batches of 100 candidates to speed up the process (2,500×600 bp instead of 2,500,000×500 bp). After the sequences were aligned, 500 bp windows from these 600 bp fragment alignments were extracted. Altogether, 2,558,882 fragment alignments were checked for their resolution capacity, i.e. usability in a SLST scheme for *P. acnes*.

**Figure 2:**
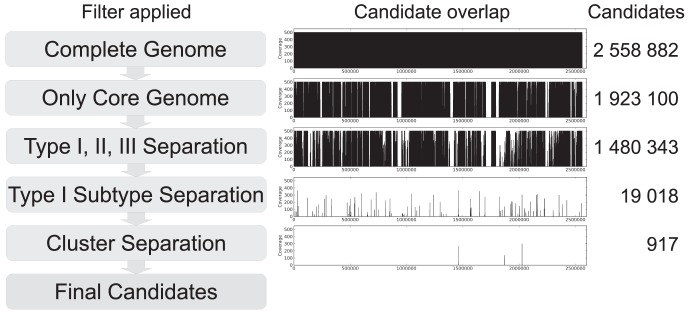
Strategy for the identification of SLST candidates in *P. acnes*. The first column shows the applied filter. The second column indicates the location of the remaining candidate fragments across the genome. The third column gives the number of remaining candidates.

The initial pool of fragments was 2,558,882, covering the complete genome. We then filtered the alignments of each of these fragments with a custom-made Python script. This filtering first removed all fragments with less than 86 aligned sequences. The remaining 1,923,100 fragments corresponded to the core genome (Figure 2). These were then tested for inconsistencies between the three major clades of *P. acnes* (types I, II, III). A total of 1,480,343 windows were then tested against strains clustering in the subtypes IA_1_, IA_2_, IB, or IC of type I, i.e.. This subtype filtering reduced the number of usable fragments to 19,018. Next, differentiation of six clusters within type IA in the reference tree was introduced as a requirement for an acceptable typing scheme. These clusters correspond to the designated letters A, B, C, D, E and F in the resulting SLST scheme (all traditionally typed IA). After this filtering, 917 SLST candidate fragments that could resolve the major clades, the subtypes of type I strains, and the six clusters within type IA remained. A substantial proportion of the 917 fragments were overlapping. All overlapping sequences (seen as three spikes in Figure 2) were merged into the final three candidates of which one was selected based on manual inspection.

### DNA extraction, PCR, Sequencing, and data analysis

To test the applicability of the final SLST candidate, primers targeting conserved regions flanking the target sequence were developed. The forward and reverse primer sequences used to target the SLST fragment were 5'-CAGCGGCGCTGCTAAGAACTT-3' and 5'-CCGGCTGGCAAATGAGGCAT-3', respectively.

A total of 77 strains covering the known diversity of *P. acnes* were used for validation of the primer pair (Table S1). DNA was extracted from isolates grown on 5% blood agar (Statens Serum Institut, Copenhagen, Denmark) for 48 hours in an anaerobic chamber. Using a 1-µl inoculation loop, colonies were collected from the agar plate and suspended in PCR-grade water. A volume of 20 µl of this suspension was mixed with 80 µl of 0.05 M NaOH and incubated for 45 minutes at 60°C. After incubation, 9.2 µl of 1 M Tris-HCl, pH 7, was added. This crude DNA preparation was diluted 100 times for PCR analysis. All PCR reactions were carried out in a 25 µl volume containing 10 µl 5′-PRIME Hotmastermix (5 PRIME, Hamburg, Germany), 3 µl of each primer (10 µM), 4 µl of PCR grade H_2_O, and 5 µl of diluted DNA. Amplification was achieved with an initial cycle of 40 seconds of denaturation at 96°C and 35 cycles of 35 seconds at 94°C for denaturation, 40 seconds at 55°C for annealing, and 40 seconds at 72°C for extension with a final extension step at 72°C for 7 minutes. Purified PCR products were Sanger sequenced from both directions at GATC Biotech, Konstanz, Germany, using standard procedures. Data analyses were carried out using MEGA.

Untrimmed sequences of approximately 600 bp were edited, aligned, and trimmed according to SLST sequences extracted from available *P. acnes* genomes. The trimmed sequences were assigned to SLST type according to the typing scheme.

### Pyrosequencing and processing of data

One sample from eight different sites of the facial skin and oral mucosa were collected from a healthy 26-year-old male with no history of acne or recent antibiotic treatment. Using a sterile cotton swap soaked in PCR grade water, symmetric samples were collected from a 2×2 cm area from the left and right side of the forehead, cheek, nose, and the buccal oral mucosa. Swabs were immediately transferred to a 1.5 ml Eppendorf tube containing 750 µl PowerLyzer PowerSoil Bead Solution (MO-BIO, Carlsbad, CA). Samples were vortexed for 2 minutes to ensure the release of bacteria to the suspension, which was then transferred to a PowerLyzer Glass Bead Tube (MO-BIO, Carlsbad, CA). DNA was extracted with the PowerLyzer PowerSoil DNA Isolation Kit (MO-BIO, Carlsbad, CA) according to the manufacturer's instructions. The SLST target sequence was amplified using fusion primers for the lib-L kit with barcodes attached to the forward primer (Table S3). PCR reaction mixtures were made in a total volume of 25 µl and comprised of 5 µl of DNA sample, 2.5 µl AccuPrime PCR Buffer II (Invitrogen), 1.5 µl of each primer (10 µM) (DNA Technology), 0.15 µl AccuPrime Taq DNA Polymerase High Fidelity (Invitrogen), and 14.35 µl of PCR grade water. PCR was performed using the following cycle conditions: an initial denaturation at 95°C for 2 minutes, followed by 30 cycles of denaturation at 95°C for 20 seconds, annealing at 55°C for 20 seconds, elongation at 72°C for 15 seconds, and then a final elongation step at 72°C for 3 minutes. Two PCR reactions were performed for all samples. The PCR products were verified on an agarose gel, pooled, and purified using the NucleoSpin Extract Kit (Macherey-Nagel). The concentration of the purified PCR product was measured with a NanoDrop 2000 spectrophotometer (Thermo Scientific). The SLST amplicons were sequenced unidirectionally from the forward primer end on a 454 Genome Sequencer FLX+ platform at GATC Biotech AG (Konstanz, Germany).

**Figure 3:**
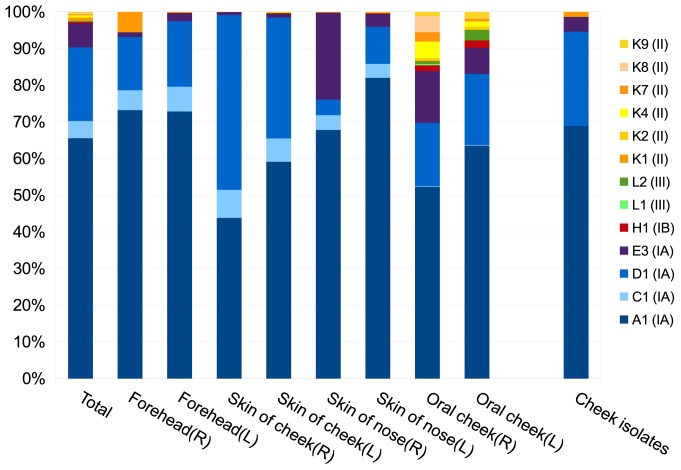
Distribution of *P. acnes* clones on skin and mucosal sites of a healthy subject revealed by application of the SLST scheme to a pyrosequencing-based analysis of complex microbiotas. Identified SLST types are color-coded. Major types IA_1_, IB, II and III are depicted in blue, red, yellow, and green and nuances thereof, respectively. The rightmost column indicates the SLST type distribution of 74 *P. acnes* isolates from a parallel sample of the skin of the cheek of the same subject. SLSTs represented by less than 0.03% of reads and reads that remained unassigned are not shown. See table S2 for distribution of all reads.

The initial processing of the data was made with the pyronoise (shhh.flows) implementation in Mothur v. 1.33.0 [24]. The reads were then assigned to STs using the best hit in a blastn comparison to the SLST database (Figure 3). The best hit was evaluated on the average of coverage and identity, and all hits lower than 99.5% were discarded as unassigned (< 9% of reads). Reads with two or more identical best hits, remained unassigned.

### Typing of *P. acnes* isolates

A swab of facial skin collected in parallel with the samples used for DNA extraction was suspended in sterile 0.85% saline and plated in 2-fold dilutions on 5% blood agar. After anaerobic incubation for four days 100 *P. acnes*-suspect colonies were subcultivated to purity and their identity tested using bacteriological procedures [15]. A total of 74 isolated confirmed as *P. acnes* were then subjected to SLST as described above.

## Results

### Identification of genomic loci suitable for SLST

The genome mining strategy resulted in the identification of three genomic loci suitable for SLST. The three regions are all found within a stretch of 600 Kb in the genome (Figure 1). Region 1 spans 881 bp and is found at nucleotide positions 2,025,740 – 2,026,621 (coordinates according to the KPA171202 genome sequence). This region partially overlaps with a coding sequence, PPA1871, annotated as 2',3'-cyclic-nucleotide 2'-phosphodiesterase precursor. Region 2 spans 719 bp and is found at nucleotide positions 1,867,701 – 1,868,420. This is upstream of the gene PPA1715, annotated as a hypothetical protein. Region 3 spans 819 bp and is found at nucleotide positions 1,462,788 – 1,463,607, immediately upstream of the gene PPA1340 encoding the CAMP factor 1, and partially overlapping with the flanking hypothetical gene PPA2385 specific to *P. acnes*.

We decided to use fragment 3, as only single nucleotide gaps were observed in some genomes, whereas alignments of the other two fragments showed gaps of up to 10 bp. By stepwise removal of nucleotides from both ends we identified a DNA sequence of 484 bp that was able to resolve all the phylogenetic clades found in our reference tree. In the following, this sequence is designated the “SLST target sequence”.

Two conserved flanking regions 85 bp downstream and 45 bp upstream of the SLST target sequence were identified as suitable PCR primers (Table S3), which resulted in an amplicon of 612 bp.

Reference sequences for alignment and trimming are found at the web-interface typing tool at http://medbac.dk/slst/pacnes. The interface allows any number of strains to be typed in batches. The sequences do not have to be trimmed, as long as the sequence is complete, and may be in reverse complement form.

### Application of the new SLST scheme

**Figure 4:**
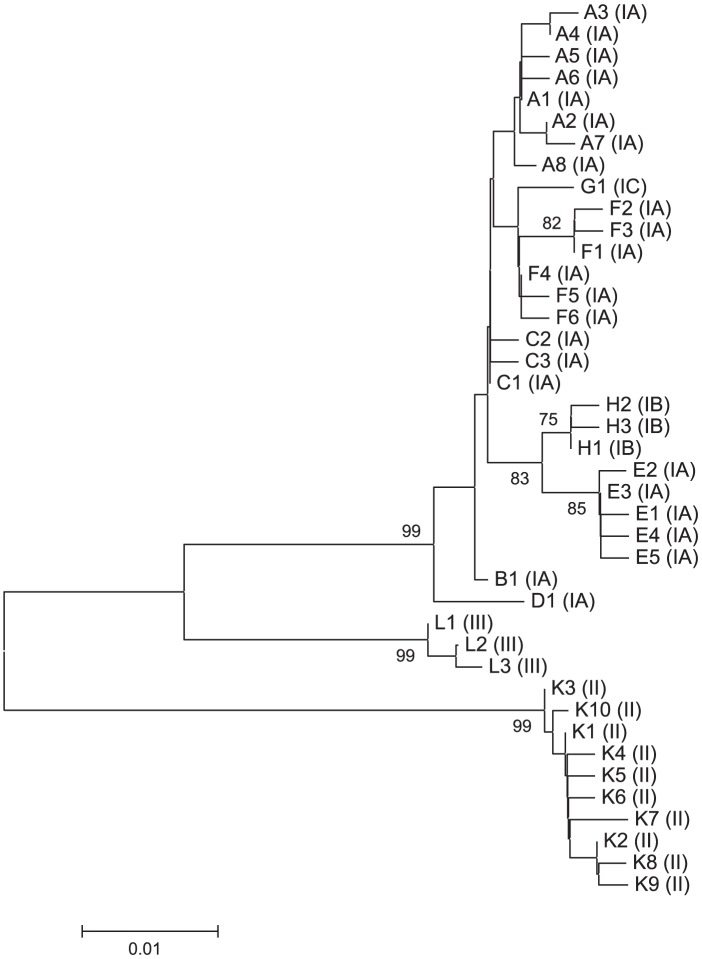
Minimum evolution tree generated from SLST target sequences of all 41 identified SLST types. Bootstrap values based on 500 replications and higher than 75% are shown.

Among 187 strains previously typed with MLST9 to represent 75 ST, 41 SLST types were identified (Table S1). A total of 80 polymorphic sites were observed in these 41 SLSTs. Phylogenetic analysis of the 41 distinct sequences revealed clustering concordant with that of the core genome reference tree (Figures 4 and 1). The chosen naming of the sequence types includes a number combined with a letter reflecting the phylogenetic relationship of strains (Figure 1). To make the transition from older schemes to the SLST scheme easier, we have appended the corresponding MLST9 type to the SLST type designations.

Overall, there is congruency between MLST9 types and the new SLST types (Figure 1). The clonal clusters (CCs) identified by eBurst analysis of MLST9 allelic profiles [17] are congruent with the letters assigned by the SLST, except for CC28 (HL025PA1 and 30.2.L1), which spans both D1 (IA) and F1-F6 (IA) (Figure 1). This phylogenetic ambiguity of CC28 is resolved neither by the MLST9 scheme nor by the MLST8 scheme reported by [19], which includes the two strains in CC-4. From the reference tree it is evident that D1 (IA) strains belong to a distinct cluster, which is detected only by the SLST.

### Species-specificity of the SLST target sequence

Alignment of the SLST target locus against the NCBI database using blastn identified 90 *P. acnes* genomes that contained this sequence, and consequently could be assigned to an SLST type (Table S1). Homologous sequences were not detected in any other bacteria represented in the NCBI database. A total of 12 of the 90 *P. acnes* genomes identified by the blastn search were not included in our phylogenetic analysis. Two were recently added to the *P. acnes* databases, i.e. the strains HL042PA3 [25] and DSM 1897. The following strains were previously identified as *P. acnes* by [19]: 409-HC1, 434-HC2, 5U42AFAA and CC003-HC2. The remaining six strains have not been assigned to species level: KPL1847, KPL1849, KPL1854, KPL2003, KPL2008, KPL2009. All of these strains cluster with *P. acnes* in a tree based on concatamers of the MLST9 genes (data not shown).

### Mapping of *P. acnes* populations in samples of complex human microbiotas

To test if the SLST scheme presented here is applicable to analysis of the population diversity of *P. acnes* in metagenomic studies, samples from eight different skin and mucosal sites (symmetric samples from the left and right side of the forehead, cheek, nose, and mouth) were collected and used to map the diversity of the *P. acnes* population. After processing, a total of 183,786 high quality reads were obtained. SLST analysis of the sequences showed that each sample contained from 4 to 13 SLTS types. Overall, the skin sample sites were dominated by *P. acnes* SLST type A1 (IA), followed by types D1 (IA), C1 (IA) and E3 (IA). Combined, these four SLSTs represented approximately 95% of the population (Figure 3). In contrast, the oral mucosal samples were characterised by more SLSTs representing clusters H, K and L (corresponding to phylotypes IB, II and III, respectively) and small proportions of C1 (IA). Comparison of the four anatomical sites sampled from the left and right sides of the same face show a clear symmetric distribution of clones. A total of 5,408 (3%) reads had two or more identical best hits and remained unassigned. Another 21,356 (11.6%) reads were unassigned due to low hit scores.

To test if reads that remained unassigned due to low hit scores represented sequence errors or new STs, 74 *P. acnes* isolates collected in parallel from the same cheek as the samples analysed by sequence-based analysis were subjected to SLST analysis. The results did not identify new types and confirmed the type distribution obtained in the sequence-based population analysis (Figure 3). Thus, it is conceivable that the unassigned reads represented sequence errors.

The sequence data have been submitted to the EMBL databases under accession No. PRJEB5434.

## Discussion

The considerable genetic diversity that may exist within bacterial species is often reflected in differences in pathogenic potential or ecological preferences. Distinct habitats may even be colonized by mixed and frequently changing populations of clones belonging to the same species [26]–[28]. To unravel such differences and their epidemiological implications, DNA-based typing methods such as MLST have been developed for most important human pathogens (www.pubmlst.org; www.mlst.net). MLST is usually uncomplicated when applied to bacterial isolates, but is time-consuming and expensive. In addition, MLST is not applicable to metagenomic studies because of the requirement to combine sequences of usually seven gene loci. Furthermore, available sequencing platforms so far lacked sufficient resolution to disclose diversity beyond the species level. Here, we present a strategy for development of single-locus typing that may be applied both to bacterial isolates and to complex microbiomes, and demonstrate its validity when applied to *P. acnes*.

One potential limitation of SLST is that it may reduce the resolution relative to MLST. However, using a genome mining strategy that may be applied to all bacteria for which sufficient genome sequences are available, we identified a 484 bp sequence that was able to resolve the vast majority of *P. acnes* clusters identified by our 9-gene MLST and even, in some cases, improve it. The second inherent limitation of SLTS is that it requires that the target species has a basically clonal population structure, because recombination will potentially blur typing results based on a single locus. Based on demonstrated strong linkage disequilibrium within allelic profiles, we previously concluded that *P. acnes* meets this criterion [15], [17]. This was confirmed by the demonstration in this study that SLST types and a tree generated on the basis of the SLST sequences without exception were congruent with lineages and clustering patterns in a phylogenetic tree based on core genome sequences of 86 *P. acnes* strains. The overall congruency between the MLST9 and SLST in an additional 187 strains further supported the conclusion that recombination affecting the target DNA sequence is rare if not non-existent.

One striking inconsistency between both MLST8 and MLST9 and results of SLST was observed. It is evident from the reference tree that the two *P. acnes* strains HL025PA1 and 30.12.L1 form a distinct cluster. Nevertheless, e-Burst analysis based on both of the two MLST schemes clustered these strains with a relatively distant group of strains [17], [19]. In contrast, the SLST scheme identified the strains as type D1 (IA) representing a distinct linage (Figure 2).

Ribotyping has been suggested as an inexpensive and efficient way of typing *P. acnes*, in particular as a tool for metagenomic analyses of the skin microbiome [20]. However, ribotyping does not discriminate the major clusters of *P. acnes*, as shown in our reference phylogenetic tree based on core genome sequences (Figure 1). For example, ribotypes 1 and 5 are present across different clusters and even across the major clades. In addition, no public *P. acnes* ribotype database is available.

We applied our SLST in a proof-of-concept analysis of the *P. acnes* population on skin and oral mucosa using a metagenomic approach. The 454 Titanium FLX+ sequencing platform used provides reads of sufficient length and quality to cover our target sequence. The inherent problems of chimeric sequences and sequencing errors resulted in 11.6% of 183,778 sequences being distinct from already identified types. We therefore used a clustering approach with very high cut off values. We find it important that new SLSTs found with this approach must be verified by high-quality Sanger-sequencing of isolated strains.

The result of our metagenomic analysis demonstrated that a single individual may be simultaneously colonized by nearly all phylogenetic clades of *P. acnes*, and that some of these may show distinct ecological preferences. For example, more types from the K (II) and L (III) clusters were found in the oral samples compared to the skin samples consistent with the demonstration of these types in endodontic infections [8]. More comprehensive studies will provide valuable information on the role of *P. acnes* in health and disease, the stability of *P. acnes* populations in distinct habitats, and must be the next step in investigations of the causality of *P. acnes-*associated diseases.

The genome mining approach used in this study to identify the SLST target sequence is applicable to other species and may be applied to other scientific problems than mere typing of members of a species. Thus, the filters may be adjusted to focus on overall resolution of the entire population, or to distinguish between functionally important subpopulations. The target sequence identified in our study is located immediately upstream of the gene PPA1340, which encodes CAMP factor 1, and partially overlaps with the hypothetical genes PPA2385 and PPA2386 (Figure S1). The upstream region of the CAMP factor 1 gene is comprised of several open reading frames that may encode smaller hypothetical proteins according to the original annotation. However, the two hypothetical, and partly overlapping genes that include the SLST region (Figure S1), are not recognised by the RAST annotation. We therefor speculate that the biological significance of this region is mainly related to transcription of the CAMP factor 1. The significance of CAMP factors in *P. acnes* has been subject of some speculation. Strikingly, five paralogous genes are present in the genome of *P. acnes* [29]. Previous research has shown an abundance of CAMP1 as surface-exposed factor [19], [30], [31]. In contrast to other CAMP factors, such as CAMP2 and CAMP4, the gene encoding CAMP1 is not located on an island-like genomic region [32]. It is possible that differences in the SLST target region are important for the transcription of CAMP1 resulting in clade-specific differences.

In conclusion, the SLST scheme shows a resolution similar to that of MLST schemes based on eight or nine loci. It is feasible to apply this method in sequence-based studies of complex microbiomes. As a proof of concept we have demonstrated that a minimum of six distinct types of *P. acnes* may coexist on the skin and oral mucosa of a healthy subject. We have implemented an SLST typing database for *P. acnes* on http://medbac.dk/slst/pacnes and propose that the presented method for identifying SLST target loci can be applied to other species with a clonal population structure.

## Supporting Information

Figure S1 **A**: Original annotation of the KPA121702 strain surrounding the SLST fragment. **B**: Alignment of sequences of all known STs in the new SLST scheme.
(PDF)

Table S1 (DOCX)

Table S2 (DOCX)

Table S3 (DOCX)
