# Supplementary figures and images for: A Novel High-Resolution Single Locus Sequence Typing Scheme for Mixed Populations of Propionibacterium acnes In Vivo
Source: PLoS One. 2014 Aug 11;9(8):e104199. doi: 10.1371/journal.pone.0104199 (PMC4128656; doi:10.1371/journal.pone.0104199)

A

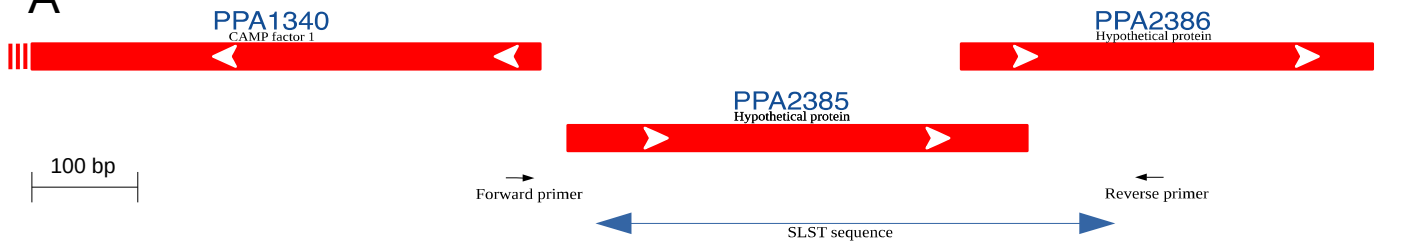

B

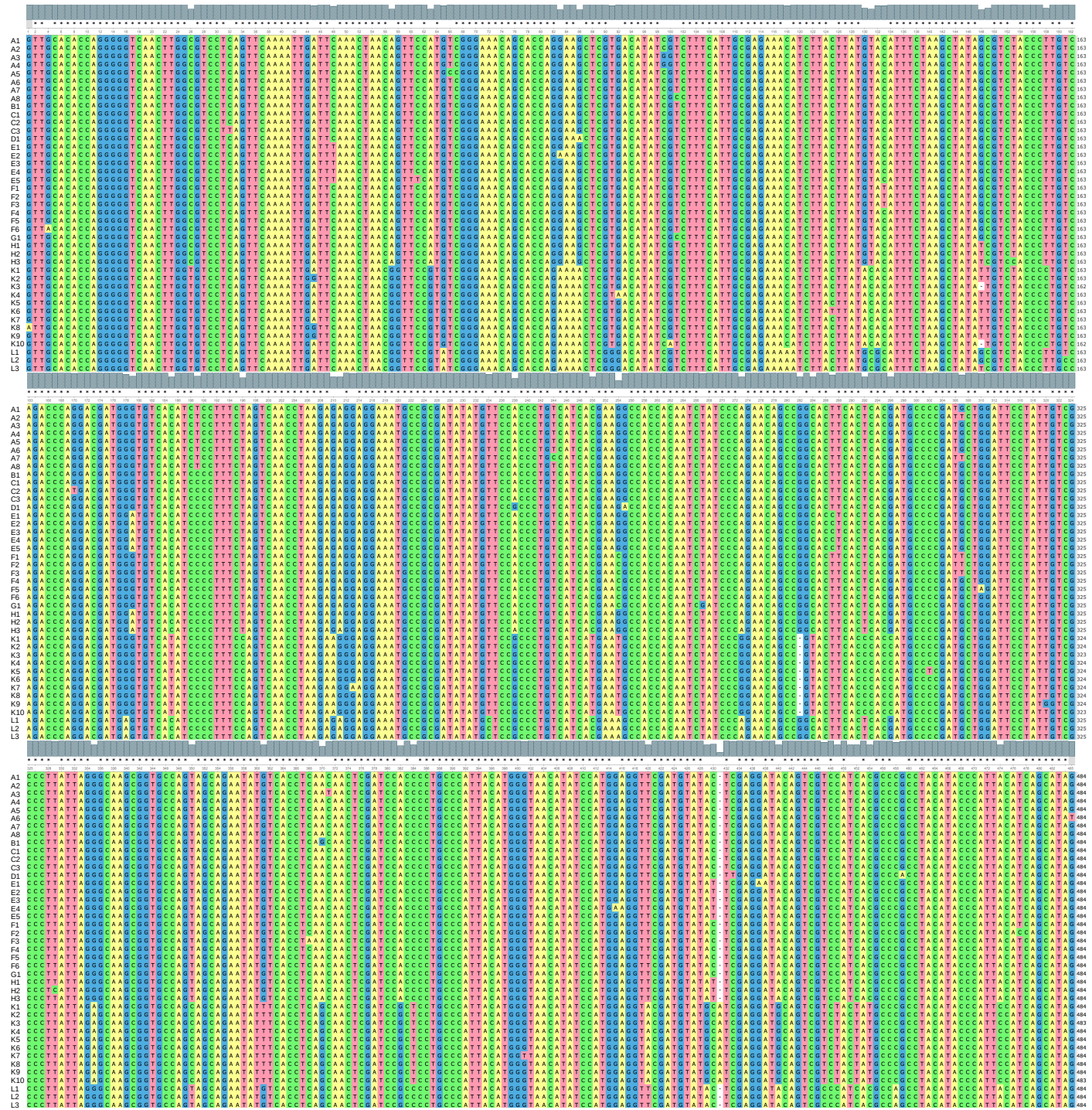

Supplement: Figure S1 — A: Original annotation of the KPA121702 strain surrounding the SLST fragment. B: Alignment of sequences of all known STs in the new SLST scheme. (PDF) [file pone.0104199.s001.pdf]
