# Supplementary material for: A Novel High-Resolution Single Locus Sequence Typing Scheme for Mixed Populations of Propionibacterium acnes In Vivo
Source: PLoS One. 2014 Aug 11;9(8):e104199. doi: 10.1371/journal.pone.0104199 (PMC4128656; doi:10.1371/journal.pone.0104199)
Supplement: Table S1 — (DOCX) [file pone.0104199.s002.docx]

**Table S1. List of 188 *Propionibacterium acnes* strains with information of the type assignments obtained by SLST, MLST9, MLST8 and ribotyping.**

| **Strain** | **SLST** | **Traditional type** | **MLST9 CC** | **MLST9** | **MLST8 CC** | **MLST8** | **Ribotyping*** | **Accesion No.** | **Status** | **Country** |
| --- | --- | --- | --- | --- | --- | --- | --- | --- | --- | --- |
| 32.1.L2 | A1 | IA | CC18 | ST18 | CC-1 | ST-1 | - |  | Contigs | Denmark |
| 37.1.L1 | A1 | IA | CC18 | ST13 | CC-1 | ST-1 | - |  | Contigs | Denmark |
| 43.1.L1 | A1 | IA | CC18 | ST12 | CC-1 | ST-1 | - |  | Contigs | Denmark |
| 44.1.L1 | A1 | IA | CC18 | ST90 | CC-1 | ST-1 | - |  | Contigs | Denmark |
| 46.1.L1 | A1 | IA | CC18 | ST18 | CC-1 | ST-1 | - |  | Contigs | Denmark |
| 48.1.L1 | A1 | IA | CC18 | ST29 | CC-1 | ST-1 | - |  | Contigs | Denmark |
| 49.1.L1 | A1 | IA | CC18 | ST18 | CC-1 | ST-1 | - |  | Contigs | Denmark |
| 50.1.L1 | A1 | IA | CC18 | ST18 | - | - | - |  | Contigs | Denmark |
| 52.1.L4 | A1 | IA | CC18 | ST18 | CC-1 | ST-1 | - |  | Contigs | Denmark |
| DSM 1897 | A1 | IA | CC18 | ST18 | CC-1 | ST-1 | - | [PRJNA169817](http://www.ncbi.nlm.nih.gov/bioproject/PRJNA169817) | Contigs | USA |
| HL005PA2 | A1 | IA | CC18 | ST12 | CC-1 | ST-15 | 1 | [PRJNA181476](http://www.ncbi.nlm.nih.gov/bioproject/PRJNA181476) | Contigs | USA |
| HL005PA3 | A1 | IA | CC18 | ST12 | CC-1 | ST-16 | 1 | [PRJNA181475](http://www.ncbi.nlm.nih.gov/bioproject/PRJNA181475) | Contigs | USA |
| HL013PA2 | A1 | IA | CC18 | ST18 | CC-1 | ST-1 | 1 | [PRJNA181450](http://www.ncbi.nlm.nih.gov/bioproject/PRJNA181450) | Contigs | USA |
| HL020PA1 | A1 | IA | CC18 | ST77 | CC-1 | ST-9 | 1 | [PRJNA181451](http://www.ncbi.nlm.nih.gov/bioproject/PRJNA181451) | Contigs | USA |
| HL027PA2 | A1 | IA | CC18 | ST12 | CC-1 | ST-1 | 1 | [PRJNA181456](http://www.ncbi.nlm.nih.gov/bioproject/PRJNA181456) | Contigs | USA |
| HL063PA1 | A1 | IA | CC18 | ST18 | CC-1 | ST-1 | 1 | [PRJNA181447](http://www.ncbi.nlm.nih.gov/bioproject/PRJNA181447) | Contigs | USA |
| HL087PA2 | A1 | IA | CC18 | ST18 | CC-1 | ST-1 | 1 | [PRJNA181460](http://www.ncbi.nlm.nih.gov/bioproject/PRJNA181460) | Contigs | USA |
| HL096PA3 | A1 | IA | CC18 | ST18 | CC-1 | ST-8 | 1 | [PRJNA181415](http://www.ncbi.nlm.nih.gov/bioproject/PRJNA181415) | Contigs | USA |
| J165 | A1 | IA | CC18 | ST18 | CC-1 | ST-1 | 1 | [PRJNA46203](http://www.ncbi.nlm.nih.gov/bioproject/PRJNA46203) | Contigs | USA |
| P. sp. 5_U_42AFAA | A1 | IA | CC18 | ST29 | CC-1 | ST-1 | 1 | [PRJNA75117](http://www.ncbi.nlm.nih.gov/bioproject/PRJNA75117) | Contigs | Canada |
| P. sp. KPL2003 | A1 | IA | CC18 | ST18 | CC-1 | ST-1 | - | [PRJNA224278](http://www.ncbi.nlm.nih.gov/bioproject/PRJNA224278) | Contigs | USA |
| 12.1.L1 | A1 | IA | CC18 | ST18 | - | - | - |  | Genome | Denmark |
| 12.1.R1 | A1 | IA | CC18 | ST20 | CC-1 | ST-20 | - |  | Genome | Denmark |
| 15.2.L1 | A1 | IA | CC18 | ST29 | CC-1 | ST-1 | - |  | Genome | Denmark |
| 266 | A1 | IA | CC18 | ST18 | CC-1 | ST-20 | 1 | [PRJNA162059](http://www.ncbi.nlm.nih.gov/bioproject/PRJNA162059) | Genome | Germany |
| 1.4.L1 | A1 | IA | CC18 | ST18 | - | - | - |  | PCR | Denmark |
| 12.1.A1 | A1 | IA | CC18 | ST18 | - | - | - |  | PCR | Denmark |
| 13.1.R1 | A1 | IA | CC18 | ST18 | - | - | - |  | PCR | Denmark |
| 18.1.R1 | A1 | IA | CC18 | ST23 | - | - | - |  | PCR | Denmark |
| 19.1.R1 | A1 | IA | CC18 | ST21 | - | - | - |  | PCR | Denmark |
| 2.4.L1 | A1 | IA | CC18 | ST18 | - | - | - |  | PCR | Denmark |
| 20.2.A1 | A1 | IA | CC18 | ST15 | - | - | - |  | PCR | Denmark |
| 21.1.A1 | A1 | IA | CC18 | ST16 | - | - | - |  | PCR | Denmark |
| 23.1.L1 | A1 | IA | CC18 | ST25 | - | - | - |  | PCR | Denmark |
| 26.2.A1 | A1 | IA | CC18 | ST17 | - | - | - |  | PCR | Denmark |
| 27.1.R1 | A1 | IA | CC18 | ST29 | - |  | - |  | PCR | Denmark |
| 32.1.R1 | A1 | IA | CC18 | ST18 | - | - | - |  | PCR | Denmark |
| 33.1.A1 | A1 | IA | CC18 | ST12 | - | - | - |  | PCR | Denmark |
| 34.2.A1 | A1 | IA | CC18 | ST18 | - | - | - |  | PCR | Denmark |
| 35.1.R1 | A1 | IA | CC18 | ST18 | - | - | - |  | PCR | Denmark |
| 37.1.A1 | A1 | IA | CC18 | ST18 | - | - | - |  | PCR | Denmark |
| 37.2.L1 | A1 | IA | CC18 | ST18 | - | - | - |  | PCR | Denmark |
| 38.1.R1 | A1 | IA | CC18 | ST18 | - | - | - |  | PCR | Denmark |
| 4.4.L1 | A1 | IA | CC18 | ST22 | - | - | - |  | PCR | Denmark |
| 4.4.R1 | A1 | IA | CC18 | ST24 | - | - | - |  | PCR | Denmark |
| 4.6.L1 | A1 | IA | CC18 | ST18 | - | - | - |  | PCR | Denmark |
| 40.1.A1 | A1 | IA | CC18 | ST18 | - | - | - |  | PCR | Denmark |
| 40.1.L1 | A1 | IA | CC18 | ST14 | - | - | - |  | PCR | Denmark |
| 5.1.A1 | A1 | IA | CC18 | ST18 | - | - | - |  | PCR | Denmark |
| 6.2.A1 | A1 | IA | CC18 | ST18 | - | - | - |  | PCR | Denmark |
| CCUG 1794 | A1 | IA | CC18 | ST18 | - | - | - |  | PCR | UK |
| CCUG 33192 | A1 | IA | CC18 | ST18 | - | - | - |  | PCR | Sweden |
| CCUG 34938 | A1 | IA | CC18 | ST19 | - | - | - |  | PCR | Sweden |
| CCUG 38203 | A1 | IA | CC18 | ST18 | - | - | - |  | PCR | Norway |
| CCUG 38584 | A1 | IA | CC18 | ST20 | - | - | - |  | PCR | USA |
| NCTC 737 | A1 | IA | CC18 | ST18 | - | - | - |  | PCR | Uk |
| 40.1.R1 | A1 | IA | Singleton | ST7 | - | - | - |  | PCR | Denmark |
| CCUG 50480 | A1 | IA | Singleton | ST6 | - | - | - |  | PCR | Sweden |
| HL002PA2 | A2 | IA | CC18 | ST59 | CC-1 | ST-1 | 1 | [PRJNA181481](http://www.ncbi.nlm.nih.gov/bioproject/PRJNA181481) | Contigs | USA |
| HL002PA3 | A2 | IA | CC18 | ST59 | CC-1 | ST-1 | 1 | [PRJNA181454](http://www.ncbi.nlm.nih.gov/bioproject/PRJNA181454) | Contigs | USA |
| HL036PA1 | A2 | IA | CC18 | ST8 | CC-1 | ST-1 | 532 | [PRJNA181448](http://www.ncbi.nlm.nih.gov/bioproject/PRJNA181448) | Contigs | USA |
| HL036PA2 | A2 | IA | CC18 | ST8 | CC-1 | ST-1 | 532 | [PRJNA181457](http://www.ncbi.nlm.nih.gov/bioproject/PRJNA181457) | Contigs | USA |
| HL036PA3 | A2 | IA | CC18 | ST8 | CC-1 | ST-1 | 1 | [PRJNA181435](http://www.ncbi.nlm.nih.gov/bioproject/PRJNA181435) | Contigs | USA |
| HL046PA2 | A2 | IA | CC18 | ST59 | CC-1 | ST-1 | 1 | [PRJNA181458](http://www.ncbi.nlm.nih.gov/bioproject/PRJNA181458) | Contigs | USA |
| China 7.1 | A2 | IA | CC18 | ST8 | - | - | - |  | PCR | China |
| China 4.1 | A2 | IA | Singleton | ST9 | - | - | - |  | PCR | China |
| 26.1.L1 | A3 | IA | CC18 | ST18 | CC-1 | ST-1 | - |  | Contigs | Denmark |
| 51.1.L11 | A3 | IA | CC18 | ST18 | CC-1 | ST-1 | - |  | Contigs | Denmark |
| 24.1.L1 | A3 | IA | CC18 | ST18 | - | - | - |  | PCR | Denmark |
| 26.2.R1 | A3 | IA | CC18 | ST18 | - | - | - |  | PCR | Denmark |
| 34.2.R1 | A4 | IA | CC18 | ST11 | - | - | - |  | PCR | Denmark |
| C1 | A5 | IA | Singleton | ST91 | CC-3 | ST-115 | 1 | [PRJNA176501](http://www.ncbi.nlm.nih.gov/bioproject/PRJNA176501) | Genome | Japan |
| China 2.1 | A5 | IA | Singleton | ST26 | - | - | - |  | PCR | China |
| HL072PA1 | A6 | IA | CC18 | ST18 | CC-1 | ST-1 | 5 | [PRJNA181468](http://www.ncbi.nlm.nih.gov/bioproject/PRJNA181468) | Contigs | USA |
| HL072PA2 | A6 | IA | CC18 | ST18 | CC-1 | ST-1 | 5 | [PRJNA181428](http://www.ncbi.nlm.nih.gov/bioproject/PRJNA181428) | Contigs | USA |
| China 8.1 | A7 | IA | CC18 | ST10 | - | - | - |  | PCR | China |
| 14.1.L1 | A8 | IA | CC18 | ST5 | - | - | - |  | PCR | Denmark |
| FZ1/2/0 | B1 | IA | Singleton | ST92 | CC-3 | ST-31 | - | [PRJNA192733](http://www.ncbi.nlm.nih.gov/bioproject/PRJNA192733) | Contigs | Hungary |
| HL078PA1 | B1 | IA | Singleton | ST89 | CC-3 | ST-14 | 1 | [PRJNA181469](http://www.ncbi.nlm.nih.gov/bioproject/PRJNA181469) | Contigs | USA |
| HL007PA1 | C1 | IA | CC3 | ST3 | CC-3 | ST-3 | 4 | [PRJNA181446](http://www.ncbi.nlm.nih.gov/bioproject/PRJNA181446) | Contigs | USA |
| HL038PA1 | C1 | IA | CC3 | ST3 | CC-3 | ST-10 | 4 | [PRJNA181440](http://www.ncbi.nlm.nih.gov/bioproject/PRJNA181440) | Contigs | USA |
| HL043PA1 | C1 | IA | CC3 | ST58 | CC-3 | ST-3 | 5 | [PRJNA181426](http://www.ncbi.nlm.nih.gov/bioproject/PRJNA181426) | Contigs | USA |
| HL043PA2 | C1 | IA | CC3 | ST58 | CC-3 | ST-3 | 5 | [PRJNA181449](http://www.ncbi.nlm.nih.gov/bioproject/PRJNA181449) | Contigs | USA |
| HL083PA1 | C1 | IA | CC3 | ST3 | CC-3 | ST-3 | 1 | [PRJNA181465](http://www.ncbi.nlm.nih.gov/bioproject/PRJNA181465) | Contigs | USA |
| HL096PA2 | C1 | IA | CC3 | ST3 | CC-3 | ST-3 | 5 | [PRJNA181416](http://www.ncbi.nlm.nih.gov/bioproject/PRJNA181416) | Contigs | USA |
| HL099PA1 | C1 | IA | CC3 | ST3 | CC-3 | ST-3 | 4 | [PRJNA181418](http://www.ncbi.nlm.nih.gov/bioproject/PRJNA181418) | Contigs | USA |
| SK182 | C1 | IA | CC3 | ST3 | Singleton | ST-29 | 1 | [PRJNA179860](http://www.ncbi.nlm.nih.gov/bioproject/PRJNA179860) | Contigs | USA |
| P. sp. KPL2009 | C1 | IA | CC3 | ST3 | CC-3 | ST-3 | - | [PRJNA224275](http://www.ncbi.nlm.nih.gov/bioproject/PRJNA224275) | Contigs | USA |
| 15.1.R1 | C1 | IA | CC3 | ST3 | CC-3 | ST-3 | - |  | Genome | Denmark |
| HL096PA1 | C1 | IA | CC3 | ST3 | CC-3 | ST-3 | - | [PRJNA198524](http://www.ncbi.nlm.nih.gov/bioproject/PRJNA198524) | Genome | USA |
| SK137 | C1 | IA | CC3 | ST3 | CC-3 | ST-18 | 1 | [PRJNA48071](http://www.ncbi.nlm.nih.gov/bioproject/PRJNA48071) | Genome | USA |
| 19.1.L1 | C1 | IA | CC3 | ST4 | - | - | - |  | PCR | Denmark |
| 3.1.A1 | C1 | IA | CC3 | ST3 | - | - | - |  | PCR | Denmark |
| 37.1.R1 | C1 | IA | CC3 | ST2 | - | - | - |  | PCR | Denmark |
| CCUG 48370 | C1 | IA | CC3 | ST3 | - | - | - |  | PCR | UK |
| 42.1.R1 | C1 | IA | Singleton | ST1 | - | - | - |  | PCR | Denmark |
| HL005PA1 | C2 | IA | CC3 | ST3 | CC-3 | ST-11 | 4 | [PRJNA181478](http://www.ncbi.nlm.nih.gov/bioproject/PRJNA181478) | Contigs | USA |
| HL045PA1 | C2 | IA | CC3 | ST3 | CC-3 | ST-17 | 4 | [PRJNA181463](http://www.ncbi.nlm.nih.gov/bioproject/PRJNA181463) | Contigs | USA |
| HL053PA1 | C2 | IA | CC3 | ST3 | CC-3 | ST-3 | 4 | [PRJNA181466](http://www.ncbi.nlm.nih.gov/bioproject/PRJNA181466) | Contigs | USA |
| HL056PA1 | C2 | IA | CC3 | ST3 | CC-3 | ST-3 | 4 | [PRJNA181445](http://www.ncbi.nlm.nih.gov/bioproject/PRJNA181445) | Contigs | USA |
| HL074PA1 | C2 | IA | CC3 | ST3 | CC-3 | ST-3 | 4 | [PRJNA181462](http://www.ncbi.nlm.nih.gov/bioproject/PRJNA181462) | Contigs | USA |
| P. sp. KPL1854 | C3 | IA | CC3 | ST3 | - | - | - | [PRJNA224280](http://www.ncbi.nlm.nih.gov/bioproject/PRJNA224280) | Contigs | USA |
| HL025PA1 | D1 | IA | CC28 | ST27 | CC-4 | ST-4 | 1 | [PRJNA181453](http://www.ncbi.nlm.nih.gov/bioproject/PRJNA181453) | Contigs | USA |
| P. sp. 409-HC1 | D1 | IA | CC28 | ST27 | - | - | - | [PRJNA67825](http://www.ncbi.nlm.nih.gov/bioproject/PRJNA67825) | Contigs | USA |
| P. sp. CC003-HC2 | D1 | IA | CC28 | ST27 | - | - | - | [PRJNA71185](http://www.ncbi.nlm.nih.gov/bioproject/PRJNA71185) | Contigs | USA |
| 30.2.L1 | D1 | IA | CC28 | ST27 | CC-4 | ST-4 | - |  | Genome | Denmark |
| CCUG 10171 | D1 | IA | CC28 | ST27 | - | - | - |  | PCR | UK |
| HL053PA2 | E1 | IA | CC31 | ST31 | CC-4 | ST-4 | 8 | [PRJNA181464](http://www.ncbi.nlm.nih.gov/bioproject/PRJNA181464) | Contigs | USA |
| HL092PA1 | E1 | IA | CC31 | ST31 | CC-4 | ST-4 | 8 | [PRJNA181431](http://www.ncbi.nlm.nih.gov/bioproject/PRJNA181431) | Contigs | USA |
| HL110PA1 | E1 | IA | CC31 | ST31 | CC-4 | ST-4 | 8 | [PRJNA181467](http://www.ncbi.nlm.nih.gov/bioproject/PRJNA181467) | Contigs | USA |
| SK187 | E2 | IA | CC28 | ST67 | Singleton | ST-19 | 3 | [PRJNA46201](http://www.ncbi.nlm.nih.gov/bioproject/PRJNA46201) | Contigs | USA |
| HL110PA2 | E3 | IA | CC31 | ST31 | CC-4 | ST-21 | 8 | [PRJNA181461](http://www.ncbi.nlm.nih.gov/bioproject/PRJNA181461) | Contigs | USA |
| 16.2.R1 | E3 | IA | Singleton | ST32 | - | - | - |  | PCR | Denmark |
| 3.6.A1 | E3 | IA | Singleton | ST31 | - | - | - |  | PCR | Denmark |
| HL086PA1 | E4 | IA | CC31 | ST31 | CC-4 | ST-4 | 8 | [PRJNA181452](http://www.ncbi.nlm.nih.gov/bioproject/PRJNA181452) | Contigs | USA |
| HL082PA1 | E5 | IA | CC31 | ST31 | CC-4 | ST-13 | 8 | [PRJNA181472](http://www.ncbi.nlm.nih.gov/bioproject/PRJNA181472) | Contigs | USA |
| HL002PA1 | F1 | IA | CC28 | ST88 | CC-2 | ST-2 | 3 | [PRJNA181433](http://www.ncbi.nlm.nih.gov/bioproject/PRJNA181433) | Contigs | USA |
| HL005PA4 | F1 | IA | CC28 | ST28 | CC-2 | ST-2 | 3 | [PRJNA181485](http://www.ncbi.nlm.nih.gov/bioproject/PRJNA181485) | Contigs | USA |
| HL013PA1 | F1 | IA | CC28 | ST87 | CC-2 | ST-2 | 3 | [PRJNA181470](http://www.ncbi.nlm.nih.gov/bioproject/PRJNA181470) | Contigs | USA |
| HL025PA2 | F1 | IA | CC28 | ST65 | CC-2 | ST-2 | 3 | [PRJNA181477](http://www.ncbi.nlm.nih.gov/bioproject/PRJNA181477) | Contigs | USA |
| HL027PA1 | F1 | IA | CC28 | ST28 | CC-2 | ST-2 | 3 | [PRJNA181434](http://www.ncbi.nlm.nih.gov/bioproject/PRJNA181434) | Contigs | USA |
| HL046PA1 | F1 | IA | CC28 | ST28 | CC-2 | ST-2 | 3 | [PRJNA181479](http://www.ncbi.nlm.nih.gov/bioproject/PRJNA181479) | Contigs | USA |
| HL050PA1 | F1 | IA | CC28 | ST28 | Singleton | ST-91 | 3 | [PRJNA181474](http://www.ncbi.nlm.nih.gov/bioproject/PRJNA181474) | Contigs | USA |
| HL050PA3 | F1 | IA | CC28 | ST28 | CC-2 | ST-2 | 3 | [PRJNA181473](http://www.ncbi.nlm.nih.gov/bioproject/PRJNA181473) | Contigs | USA |
| HL059PA1 | F1 | IA | CC28 | ST28 | CC-2 | ST-2 | 16 | [PRJNA181459](http://www.ncbi.nlm.nih.gov/bioproject/PRJNA181459) | Contigs | USA |
| HL059PA2 | F1 | IA | CC28 | ST28 | CC-2 | ST-2 | 16 | [PRJNA181429](http://www.ncbi.nlm.nih.gov/bioproject/PRJNA181429) | Contigs | USA |
| HL087PA1 | F1 | IA | CC28 | ST28 | CC-2 | ST-2 | 3 | [PRJNA181427](http://www.ncbi.nlm.nih.gov/bioproject/PRJNA181427) | Contigs | USA |
| HL087PA3 | F1 | IA | CC28 | ST28 | CC-2 | ST-2 | 3 | [PRJNA181437](http://www.ncbi.nlm.nih.gov/bioproject/PRJNA181437) | Contigs | USA |
| 25.1.R1 | F1 | IA | CC28 | ST28 | - | - | - |  | PCR | Denmark |
| P.acn33 | F1 | IA | Singleton | ST86 | CC-2 | ST-2 | 3 | [PRJNA80745](http://www.ncbi.nlm.nih.gov/bioproject/PRJNA80745) | Genome | UK |
| HL067PA1 | F2 | IA | CC28 | ST64 | CC-2 | ST-24 | 3 | [PRJNA181482](http://www.ncbi.nlm.nih.gov/bioproject/PRJNA181482) | Contigs | USA |
| HL083PA2 | F3 | IA | CC28 | ST28 | CC-2 | ST-2 | 3 | [PRJNA181430](http://www.ncbi.nlm.nih.gov/bioproject/PRJNA181430) | Contigs | USA |
| HL030PA2 | F4 | IA | CC28 | ST66 | Singleton | ST-22 | 3 | [PRJNA181436](http://www.ncbi.nlm.nih.gov/bioproject/PRJNA181436) | Contigs | USA |
| HL063PA2 | F4 | IA | CC28 | ST70 | Singleton | ST-23 | 3 | [PRJNA181455](http://www.ncbi.nlm.nih.gov/bioproject/PRJNA181455) | Contigs | USA |
| P.acn31 | F4 | IA | CC28 | ST67 | CC-2 | ST-36 | 3 | [PRJNA80733](http://www.ncbi.nlm.nih.gov/bioproject/PRJNA80733) | Genome | UK |
| 20.2.R1 | F4 | IA | CC28 | ST30 | - | - | - |  | PCR | Denmark |
| P.acn17 | F5 | IA | CC28 | ST70 | Singleton | ST-22 | 3 | [PRJNA80735](http://www.ncbi.nlm.nih.gov/bioproject/PRJNA80735) | Genome | UK |
| HL037PA1 | F6 | IA | CC28 | ST67 | CC-2 | ST-2 | 3 | [PRJNA181471](http://www.ncbi.nlm.nih.gov/bioproject/PRJNA181471) | Contigs | USA |
| HL097PA1 | G1 | IC | Singleton | ST74 | CC-107 | ST-70 | 5 | [PRJNA181419](http://www.ncbi.nlm.nih.gov/bioproject/PRJNA181419) | Contigs | USA |
| PRP-38 | G1 | IC | Singleton | ST74 | CC-107 | ST-70 | 5 | [PRJNA180249](http://www.ncbi.nlm.nih.gov/bioproject/PRJNA180249) | Contigs | UK |
| HL030PA1 | H1 | IB | CC36 | ST36 | CC-5 | ST-5 | 1 | [PRJNA181438](http://www.ncbi.nlm.nih.gov/bioproject/PRJNA181438) | Contigs | USA |
| P. sp. KPL2008 | H1 | IB | CC36 | ST36 | CC-5 | ST-5 | - | [PRJNA224276](http://www.ncbi.nlm.nih.gov/bioproject/PRJNA224276) | Contigs | USA |
| 21.1.L1 | H1 | IB | CC36 | ST36 | CC-5 | ST-56 | - |  | Genome | Denmark |
| 6609 | H1 | IB | CC36 | ST93 | CC-5 | ST-5 | 1 | [PRJNA162137](http://www.ncbi.nlm.nih.gov/bioproject/PRJNA162137) | Genome | Hungary |
| 2.3.A1 | H1 | IB | CC36 | ST35 | - | - | - |  | PCR | Denmark |
| 21.2.A1 | H1 | IB | CC36 | ST37 | - | - | - |  | PCR | Denmark |
| 27.1.A1 | H1 | IB | CC36 | ST38 | - | - | - |  | PCR | Denmark |
| 27.1.L1 | H1 | IB | CC36 | ST40 | - | - | - |  | PCR | Denmark |
| 36.1.L1 | H1 | IB | CC36 | ST41 | - | - | - |  | PCR | Denmark |
| CCUG 36661 | H1 | IB | CC36 | ST42 | - | - | - |  | PCR | Sweden |
| CCUG 47251 | H1 | IB | CC36 | ST33 | - | - | - |  | PCR | Sweden |
| CCUG32901 | H1 | IB | CC36 | ST33 | - | - | - |  | PCR | Sweden |
| KPA171202 | H2 | IB | CC36 | ST36 | CC-5 | ST-5 | 1 | [PRJNA58101](http://www.ncbi.nlm.nih.gov/bioproject/PRJNA58101) | Genome | Germany |
| DSM16379 | H2 | IB | CC36 | ST34 | - | - | - |  | PCR | Germany |
| CCUG 48138 | H3 | IB | CC36 | ST36 | - | - | - |  | PCR | Sweden |
| HL001PA1 | K1 | II | CC53 | ST60 | CC-72 | ST-30 | 2 | [PRJNA181484](http://www.ncbi.nlm.nih.gov/bioproject/PRJNA181484) | Contigs | USA |
| HL060PA1 | K1 | II | CC53 | ST69 | CC-6 | ST-6 | 2 | [PRJNA181441](http://www.ncbi.nlm.nih.gov/bioproject/PRJNA181441) | Contigs | USA |
| HL103PA1 | K1 | II | CC53 | ST60 | CC-6 | ST-25 | 6 | [PRJNA181417](http://www.ncbi.nlm.nih.gov/bioproject/PRJNA181417) | Contigs | USA |
| 18.1.A1 | K1 | II | CC53 | ST53 | - | - | - |  | PCR | Denmark |
| 18.2.L1 | K1 | II | CC53 | ST47 | - | - | - |  | PCR | Denmark |
| China 2.3 | K1 | II | CC53 | ST49 | - | - | - |  | PCR | China |
| CCUG 33206 | K1 | II | Singleton | ST57 | - | - | - |  | PCR | Sweden |
| HL042PA3 | K2 | II | CC53 | ST73 | CC-6 | ST-7 | - | [PRJNA225886](http://www.ncbi.nlm.nih.gov/bioproject/PRJNA225886) | Contigs | USA |
| HL110PA3 | K2 | II | CC53 | ST73 | CC-6 | ST-7 | 6 | [PRJNA181443](http://www.ncbi.nlm.nih.gov/bioproject/PRJNA181443) | Contigs | USA |
| HL110PA4 | K2 | II | CC53 | ST73 | CC-6 | ST-7 | 6 | [PRJNA181442](http://www.ncbi.nlm.nih.gov/bioproject/PRJNA181442) | Contigs | USA |
| CCUG 6528 | K2 | II | CC53 | ST52 | - | - | - |  | PCR | Sweden |
| 7.1.L1 | K2 | II | Singleton | ST50 | - | - | - |  | PCR | Denmark |
| 39.3.R1 | K3 | II | Singleton | ST56 | - | - | - |  | PCR | Denmark |
| HL050PA2 | K4 | II | CC53 | ST60 | Singleton | ST-26 | 1 | [PRJNA181439](http://www.ncbi.nlm.nih.gov/bioproject/PRJNA181439) | Contigs | USA |
| P. sp. KPL1847 | K4 | II | CC53 | ST60 | - | - | - | [PRJNA224283](http://www.ncbi.nlm.nih.gov/bioproject/PRJNA224283) | Contigs | USA |
| P. sp. KPL1849 | K4 | II | CC53 | ST60 | - | - | - | [PRJNA224282](http://www.ncbi.nlm.nih.gov/bioproject/PRJNA224282) | Contigs | USA |
| 34.1.A1 | K4 | II | CC53 | ST54 | - | - | - |  | PCR | Denmark |
| CCUG 33950 | K5 | II | CC53 | ST53 |  |  | - |  | PCR | Sweden |
| HL082PA2 | K6 | II | Singleton | ST62 | CC-6 | ST-6 | 2 | [PRJNA181480](http://www.ncbi.nlm.nih.gov/bioproject/PRJNA181480) | Contigs | USA |
| CCUG 33951 | K7 | II | CC53 | ST48 | - | - | - |  | PCR | Sweden |
| J139 | K8 | II | CC53 | ST73 | CC-72 | ST-28 | 2 | [PRJNA42957](http://www.ncbi.nlm.nih.gov/bioproject/PRJNA42957) | Contigs | USA |
| 5.1.R1 | K8 | II | CC53 | ST52 | - | - | - |  | PCR | Denmark |
| CCUG 38293 | K8 | II | CC53 | ST52 | - | - | - |  | PCR | Sweden |
| CCUG 50655 | K8 | II | CC53 | ST46 | - | - | - |  | PCR | Sweden |
| ATCC 11828 | K9 | II | CC53 | ST85 | Singleton | ST-27 | 2 | [PRJNA162177](http://www.ncbi.nlm.nih.gov/bioproject/PRJNA162177) | Genome | Hungary |
| CCUG 6369 | K9 | II | CC53 | ST52 | - | - | - |  | PCR | Germany |
| PMH5 | L1 | III | Singleton | ST44 | CC-77 | ST-33 | - |  | Genome | Denmark |
| PMH7 | L1 | III | Singleton | ST44 | CC-77 | ST-33 | - |  | Genome | Denmark |
| CCUG 35547 | L1 | III | Singleton | ST44 | - | - | - |  | PCR | Sweden |
| CCUG 35749 | L2 | III | Singleton | ST43 | - | - | - |  | PCR | Norway |
| CCUG 35900 | L2 | III | Singleton | ST43 | - | - | - |  | PCR | Sweden |
| CCUG 36986 | L2 | III | Singleton | ST44 | - | - | - |  | PCR | Sweden |
| N1300842 | L3 | III | - | - | - | - | - |  | PCR | Germany |

* Data found on <http://pubmlst.org/pacnes/>
