# Supplementary material for: A Novel High-Resolution Single Locus Sequence Typing Scheme for Mixed Populations of Propionibacterium acnes In Vivo
Source: PLoS One. 2014 Aug 11;9(8):e104199. doi: 10.1371/journal.pone.0104199 (PMC4128656; doi:10.1371/journal.pone.0104199)
Supplement: Table S2 — (DOCX) [file pone.0104199.s003.docx]

**Table S2. Sequence read distribution of identified SLST types.**

| SLST | Total | Forehead (R) | Forehead (L) | Skin of cheek (R) | Skin of cheek (L) | Skin of nose (R) | Skin of nose (L) | Buccal mucosa (R) | Buccal mucosa (L) | Skin of cheek isolates |
| --- | --- | --- | --- | --- | --- | --- | --- | --- | --- | --- |
| A1 | 106,393 | 14,990 | 16,421 | 8,586 | 13,921 | 16,393 | 20,649 | 7,368 | 8,065 | 51 |
| A2 | 0 | 0 | 0 | 0 | 0 | 0 | 0 | 0 | 0 | 0 |
| A3 | 0 | 0 | 0 | 0 | 0 | 0 | 0 | 0 | 0 | 0 |
| A4 | 9 | 0 | 0 | 0 | 0 | 0 | 0 | 0 | 9 | 0 |
| A5 | 23 | 5 | 2 | 1 | 4 | 3 | 2 | 3 | 3 | 0 |
| A6 | 0 | 0 | 0 | 0 | 0 | 0 | 0 | 0 | 0 | 0 |
| A7 | 0 | 0 | 0 | 0 | 0 | 0 | 0 | 0 | 0 | 0 |
| A8 | 35 | 3 | 4 | 6 | 4 | 6 | 6 | 3 | 3 | 0 |
| B1 | 7 | 1 | 1 | 1 | 2 | 2 | 0 | 0 | 0 | 0 |
| C1 | 7,617 | 1,110 | 1,529 | 1,502 | 1,500 | 974 | 959 | 22 | 21 | 0 |
| C2 | 5 | 0 | 0 | 0 | 0 | 2 | 3 | 0 | 0 | 0 |
| C3 | 1 | 0 | 0 | 0 | 0 | 1 | 0 | 0 | 0 | 0 |
| D1 | 32,582 | 2,983 | 4,026 | 9,345 | 7,756 | 1,028 | 2,545 | 2,436 | 2,463 | 0 |
| E1 | 1 | 0 | 0 | 0 | 0 | 1 | 0 | 0 | 0 | 0 |
| E2 | 0 | 0 | 0 | 0 | 0 | 0 | 0 | 0 | 0 | 0 |
| E3 | 10,676 | 251 | 495 | 149 | 263 | 5,727 | 897 | 1,989 | 905 | 19 |
| E4 | 0 | 0 | 0 | 0 | 0 | 0 | 0 | 0 | 0 | 0 |
| E5 | 2 | 0 | 0 | 0 | 0 | 2 | 0 | 0 | 0 | 0 |
| F1 | 8 | 0 | 0 | 8 | 0 | 0 | 0 | 0 | 0 | 3 |
| F2 | 0 | 0 | 0 | 0 | 0 | 0 | 0 | 0 | 0 | 0 |
| F3 | 0 | 0 | 0 | 0 | 0 | 0 | 0 | 0 | 0 | 0 |
| F4 | 1 | 0 | 0 | 1 | 0 | 0 | 0 | 0 | 0 | 0 |
| F5 | 0 | 0 | 0 | 0 | 0 | 0 | 0 | 0 | 0 | 0 |
| F6 | 0 | 0 | 0 | 0 | 0 | 0 | 0 | 0 | 0 | 0 |
| G1 | 0 | 0 | 0 | 0 | 0 | 0 | 0 | 0 | 0 | 0 |
| H1 | 550 | 2 | 13 | 5 | 16 | 11 | 29 | 221 | 253 | 0 |
| H2 | 41 | 0 | 0 | 0 | 0 | 0 | 0 | 41 | 0 | 0 |
| H3 | 0 | 0 | 0 | 0 | 0 | 0 | 0 | 0 | 0 | 1 |
| K1 | 1,289 | 1,139 | 58 | 5 | 11 | 22 | 36 | 18 | 0 | 0 |
| K10 | 0 | 0 | 0 | 0 | 0 | 0 | 0 | 0 | 0 | 0 |
| K2 | 346 | 0 | 1 | 1 | 61 | 15 | 53 | 102 | 113 | 0 |
| K3 | 0 | 0 | 0 | 0 | 0 | 0 | 0 | 0 | 0 | 0 |
| K4 | 805 | 0 | 0 | 0 | 0 | 0 | 0 | 630 | 175 | 0 |
| K5 | 0 | 0 | 0 | 0 | 0 | 0 | 0 | 0 | 0 | 0 |
| K6 | 0 | 0 | 0 | 0 | 0 | 0 | 0 | 0 | 0 | 0 |
| K7 | 463 | 0 | 0 | 0 | 2 | 3 | 3 | 364 | 91 | 0 |
| K8 | 631 | 0 | 1 | 0 | 0 | 1 | 2 | 616 | 11 | 0 |
| K9 | 388 | 0 | 0 | 1 | 0 | 0 | 0 | 159 | 228 | 0 |
| L1 | 50 | 0 | 0 | 0 | 0 | 0 | 0 | 50 | 0 | 0 |
| L2 | 499 | 0 | 0 | 0 | 16 | 1 | 0 | 111 | 371 | 0 |
| L3 | 0 | 0 | 0 | 0 | 0 | 0 | 0 | 0 | 0 | 0 |
| Total | 183,778 | 25,478 | 24,853 | 22,063 | 26,729 | 27,991 | 27,549 | 15,477 | 13,638 | 74 |
| Unassigned multiple hits | 5,408 | 1,246 | 709 | 498 | 801 | 1 025 | 694 | 230 | 205 | 0 |
| Unassigned poor quality | 21,356 | 4,994 | 2,302 | 2,452 | 3,173 | 3,799 | 2,365 | 1,344 | 927 | 0 |
