# Supplementary material for: A Novel High-Resolution Single Locus Sequence Typing Scheme for Mixed Populations of Propionibacterium acnes In Vivo
Source: PLoS One. 2014 Aug 11;9(8):e104199. doi: 10.1371/journal.pone.0104199 (PMC4128656; doi:10.1371/journal.pone.0104199)
Supplement: Table S3 — (DOCX) [file pone.0104199.s004.docx]

| Primer ID | Sequence |
| --- | --- |
| SLST-Forward | 5'-CAGCGGCGCTGCTAAGAACTT-3' |
| SLST-Reverse | 5'-CCGGCTGGCAAATGAGGCAT-3' |
| Tag-1-Forward | 5'-CCATCTCATCCCTGCGTGTCTCCGACTCAG CGCAA CCAGCGGCGCTGCTAAGAACTT-3' |
| Tag-2-Forward | 5'-CCATCTCATCCCTGCGTGTCTCCGACTCAG TGAAG CCAGCGGCGCTGCTAAGAACTT-3' |
| Tag-3-Forward | 5'-CCATCTCATCCCTGCGTGTCTCCGACTCAG ACTTG CCAGCGGCGCTGCTAAGAACTT-3' |
| Tag-4-Forward | 5'-CCATCTCATCCCTGCGTGTCTCCGACTCAG TCACA CCAGCGGCGCTGCTAAGAACTT-3' |
| Tag-5-Forward | 5'-CCATCTCATCCCTGCGTGTCTCCGACTCAG CGTGA CCAGCGGCGCTGCTAAGAACTT-3' |
| Tag-6-Forward | 5'-CCATCTCATCCCTGCGTGTCTCCGACTCAG ACGCG CCAGCGGCGCTGCTAAGAACTT-3' |
| Tag-7-Forward | 5'-CCATCTCATCCCTGCGTGTCTCCGACTCAG CCTCT CCAGCGGCGCTGCTAAGAACTT-3' |
| Tag-8-Forward | 5'-CCATCTCATCCCTGCGTGTCTCCGACTCAG ACTCA CCAGCGGCGCTGCTAAGAACTT-3' |
| Tag-Reverse | 5'-CCTATCCCCTGTGTGCCTTGGCAGTCTCAG CCGGCTGGCAAATGAGGCAT-3' |

**Table S3. List of primers used in the study.**
